# Social Support Behaviours and Barriers in Group Online Exercise Classes for Adults Living with and beyond Cancer: A Qualitative Study

**DOI:** 10.3390/curroncol30040284

**Published:** 2023-03-28

**Authors:** Bobbie-Ann P. Craig, Meghan H. McDonough, S. Nicole Culos-Reed, William Bridel

**Affiliations:** Faculty of Kinesiology, University of Calgary, 2500 University Drive NW, Calgary, AB T2N 1N4, Canada

**Keywords:** oncology, physical activity, synchronous, supportive behaviours, instructor training

## Abstract

Social support can be facilitated through exercise programs for people living with cancer, but there is limited research on how best to foster it in online exercise oncology classes. This study examined current training that fitness professionals receive on the provision and facilitation of social support, experiences people living with cancer have with social support, and supportive behaviours and barriers for providing and obtaining support in online group exercise oncology programs in Calgary, Alberta, Canada. Guided by interpretive description methodology, training materials were reviewed, observations of fitness professional training and online exercise classes (*n =* 10) were conducted, and adults living with and beyond cancer (*n =* 19) and fitness professionals (*n =* 15) were interviewed. These data were collected from January 2021 to June 2021. Analysis of the data collected resulted in the identification of three themes: Creating a welcoming environment, helping improve exercise ability and reach goals, and learning to provide and facilitate support online. A catalogue of supportive behaviours that can help to provide and facilitate and barriers that can hinder the provision and obtaining of social support in exercise oncology classes is presented. The findings provide guidance when structuring online classes and inform developing strategies for fitness professionals to use in online classes to foster social support by considering the wants and needs of participants, facilitating support between participants with similar experiences and interests, and integrating support into physical activity.

## 1. Introduction

Group exercise oncology programs (group exercise programs for adults living with and beyond cancer) can provide opportunities to receive social support (SS) from other exercisers and fitness professionals [1]. Fitness professionals can also facilitate SS among participants, for example, by encouraging discussion [2]. Much of the research on supportive interactions in exercise oncology has examined programs delivered in person. However, many individuals are unable to access in-person programs, and online exercise delivery can address this barrier [3]. Fitness professionals can contribute to perceptions of being supported and decrease feelings of loneliness via online interactions [4], and group-based synchronous online exercise may provide opportunities for peer connection and socialization [5,6]. However, SS from peers and instructors is often diminished online as compared to in-person formats [7]. Research on how fitness professionals are trained to provide and facilitate SS, and behaviours participants find supportive in the online context is limited.

### 1.1. Theoretical Framework

This study is informed by SS theory [8], which highlights that focusing on supportive functions and behaviours is important for developing actionable strategies to improve support in programs and that SS can help individuals cope with adversity, as well as grow and thrive. SS includes interpersonal interactions or actions another person initiates or does to try to help or that may influence other people. The theory identifies four types of sources of strength support, which assist individuals coping with adversity: (1) Providing a safe haven where an individual feels comfortable and accepted, (2) providing fortification or assistance in developing strengths that aid in coping with adversity, (3) helping with reconstruction by supporting individuals to use their strengths to problem solve and cope while not dwelling on the negative, and (4) reframing adversity as less threatening or finding benefits in adversity. There are also four types of relational catalyst support, which help people grow and thrive: (1) Nurturing a desire to create and seize life opportunities by encouraging individuals to pursue challenges, (2) providing perceptual assistance in recognizing life opportunities as positive challenges, (3) facilitating preparation for engagement in life opportunities by promoting skill development, and (4) providing a launching function that enables individuals to fully engage in life opportunities.

### 1.2. Social Support in Group Exercise for Adults Living with and beyond Cancer

Group exercise oncology programs create opportunities to receive and provide SS and develop friendships with others who have cancer [1,9]. These programs bring together people who understand the cancer experience and provide the opportunity to share first-hand information [1,10]. However, being around others with cancer may be difficult as it can draw attention to one’s own morbidity and mortality [1,11]. PA contexts can be a useful bridge between these benefits and challenges as many participants appreciate being able to talk about cancer if they want, but in a context where the focus is on a positive, healthy pursuit [1,9]. These programs can also normalize cancer and be a space where individuals do not need to explain they have cancer, are not treated as a patient or victim, and can talk openly and use dark humour that might make others uncomfortable in other settings [1,12].

While most prior research has focused on establishing a connection between perceptions of feeling supported and positive outcomes, a recent review of the qualitative literature in this area identified several supportive behaviours [1]. Other exercisers can provide SS by encouraging PA engagement, recommending exercise programs, demonstrating what other people in similar circumstances can do, providing companionship, making PA fun through socializing, and supporting PA mastery by encouraging a non-competitive environment. Interacting regularly through PA provides a reason to come together, which helps build relationships. However, exercising together and the common experience of cancer are not necessarily sufficient for supportive relationships to form [2]. There is some evidence that fitness professionals can play a role in encouraging SS by facilitating interactions between participants, for example, by inviting participants to share how they are feeling [13], incorporating everyone in group activities, or inviting other people to talk if someone is dominating the conversation [2].

Fitness professionals can also directly provide SS to participants by developing relationships and having positive interactions with them [10,14]. Fitness professionals who are certified, competent, experienced, and knowledgeable about cancer can instill confidence and trust [5,10,15]. Trained, experienced professionals who have the skills to modify exercises based on individuals’ needs and preferences can help participants feel included, like classes are tailored for them and that they are not being judged [10,16]. However, there is limited research on behaviours fitness professionals can exhibit to provide and facilitate SS, particularly in the online context. This is a gap in the literature that this study will address by observing the online exercise classes, as well as how fitness professionals are trained to provide and facilitate support.

### 1.3. Online Exercise Classes for Adults Living with and beyond Cancer

Online programming helps reach participants who are unable to access in-person services [3,5] due to factors such as convenience (e.g., no commute, can take the class anywhere) and the comfort of staying at home [3,10,17]. Common barriers include poor internet access, lack of equipment, safety concerns, and decreased opportunities for SS [3,17]. Many adults living with and beyond cancer prefer in-person programs because of the social interaction and replicating social opportunities online is challenging [17]. Identifying socially supportive behaviours and barriers to SS online is an important gap that needs to be filled to improve programs.

Fitness professionals can improve participants’ perceptions of support and decrease feelings of loneliness [4]. Participants identify that having a knowledgeable fitness professional who is available and genuinely cares about their well-being is important to them [18,19]. Exercise oncology program developers and fitness professionals who work with this population have provided guidance for delivering virtual fitness classes based on their early experiences in the online environment. They suggest creating a connected community by providing time to connect before and after classes, scheduling in-person social events, organizing online forums to interact and support one another, helping clients relate to each other, building rapport, providing clear communication, listening, posing open-ended questions, pointing out how to improve rather than errors, educating, and empowering [6]. This study is needed to explore the range of SS behaviours in online classes, understand which behaviours are perceived as supportive by exercise oncology class participants, and identify barriers fitness professionals experience to providing SS online.

The need for online classes grew during the COVID-19 pandemic, as did research exploring the feasibility and effectiveness of online synchronous group exercise oncology programs (group exercise classes offered to adults living with and beyond cancer that include live instruction from a fitness professional in the online environment) [20]. One-on-one online programs have demonstrated potential to improve fatigue, perceptions of support, quality of life, loneliness and reduce anxiety/stress [20]. However, a drawback of online programs and one-on-one programs in particular, is that opportunities for SS and camaraderie with peers are reduced [5,7]. Although research on group programs is limited, there is evidence of increased PA and reduction of sedentary behaviour after participating [20], and participants have reported feelings of connection during classes even when they were required to keep their audio and video off [21]. Understanding how to enhance support in online synchronous group exercise oncology classes may help to create opportunities for SS and connection between peers [5].

### 1.4. Purpose

While it is well known SS is important for helping people cope with cancer and engage in PA behaviour, and supportive behaviours have been identified in the in person exercise oncology context, there is limited evidence regarding what behaviours are perceived as supportive by participants in the online environment. Furthermore, there is very limited instruction for how fitness professionals can provide SS online and what training they receive regarding SS in the online context. The purpose of this study was to (1) identify current practices for training fitness professionals to provide and facilitate SS, (2) understand the experiences of adults living with and beyond cancer with SS from other exercisers and fitness professionals, and (3) identify barriers and facilitators for providing (by fitness professionals) and obtaining (by adults living with and beyond cancer) SS in online exercise oncology programs.

## 2. Materials and Methods

### 2.1. Methodology

We used a qualitative case study design [22], which allows researchers to examine a specific context and phenomena of interest from multiple perspectives. In this study, we examined social support in two online exercise oncology programs. Five sources of data were collected: (1) A document review of fitness professional training materials, (2) observation of a fitness professional training session, (3) observation of online exercise oncology classes, (4) interviews with fitness professionals, and (5) interviews with exercise class participants (these sources of data are explained in detail in 2.4. Procedures). This was done in order to understand SS behaviours informed by data on how professionals are trained to use SS, how they implement that training, their perspectives on the behaviours they use, and their participants’ perspectives on supportive behaviours they experience to gain a comprehensive understanding of SS in the online context. We used interpretive description methodology, which examines applied health questions from the perspective of participant experiences interpreted using theoretical, disciplinary, and empirical knowledge [23]. This methodology allowed us to connect SS theory [8] to the online exercise oncology context and provided a framework for interpreting the data inductively while incorporating disciplinary and empirical knowledge. We also used a constructivist epistemology (beliefs about how knowledge is acquired) and a relativist ontology (assumption about the world and what is reality) [23]. Constructivism posits that human experience is socially constructed and involves multiple realities, while relativism suggests there are multiple social realities that are subjective [23].

### 2.2. The Context

This data were collected from January 2021 to June 2021. The participants in the current study had experience in one of two group online exercise oncology programs that were part of ongoing research through the Health and Wellness Lab at the University of Calgary in Calgary, Alberta, Canada. First, the Alberta Cancer Exercise (ACE) program is a hybrid effectiveness-implementation study that offers free, supervised, group-based exercise oncology classes, delivered online and in person, to individuals with any form of cancer, up to three years post-treatment completion [24]. Full details of the ACE intervention protocol [24] are available. Second, the Exercise for Cancer to Enhance Living Well (EXCEL) program is an ongoing study offering free, supervised, online group-based exercise oncology classes to individuals living in rural or remote regions across Canada with any form of cancer who are about to have, are undergoing, or have had treatment within the last five years [24]; NCT04478851]. Full details are included in the protocol [NCT04478851], and the class content, length, and program duration are similar to ACE [24]. For the current study, adults living with and beyond cancer and fitness professionals from ACE and EXCEL classes were recruited. Some participants also had experience with fitness programs outside of the university and/or in person classes prior to COVID-19. Both ACE and EXCEL use the same fitness professional training program.

### 2.3. Participants

#### 2.3.1. Fitness Professionals

Fitness professionals (i.e., instructors, people who help the instructors (moderators), and people who coordinate/plan the exercise classes) were eligible for the study if they (1) had taught or moderated an online exercise oncology class and (2) were able to participate in an interview via telephone or online (e.g., Zoom). Fifteen fitness professionals aged 21–44 years participated (see Table 1 for demographic information). All had a close friend or family member diagnosed with cancer. All were required to take the Thrive Health Services Cancer and Exercise training to deliver ACE or EXCEL classes. Eight had or were working towards a bachelor’s degree in kinesiology, four were clinical exercise physiologists, two were certified personal trainers, and one was a certified physical literacy instructor. Experience instructing online exercise oncology programs ranged from eight weeks to four years, and 10 also had experience instructing in-person exercise oncology programs (range = 1 month to 11 years). All were currently instructing or moderating an online exercise oncology class.

#### 2.3.2. Adults Living with and beyond Cancer

Adults living with and beyond cancer were eligible for the study if they (1) were 18 years of age or older, (2) fluent in English, (3) had participated in an online group exercise oncology class, and (4) were able to participate in an interview via telephone or online (e.g., Zoom). Nineteen people aged 24–74 years (see Table 1 for demographic information). Most were married or living with a spouse (78.9%), and of those, their current relationships ranged from 9 to 52 years (M = 32.7 years, SD = 10.4 years). Four of the participants’ partners typically engaged in PA with them. Most had breast (26.3%) or prostate (15.8%) cancer and were diagnosed with Stage 1 to 4 cancer. Most recent cancer diagnoses ranged from <1 to 12 years prior, three were undergoing treatment, and three had a reoccurrence or new incidence of cancer since their initial diagnosis. Participants had undergone multiple treatments, including radiation, chemotherapy, surgery, drug therapy, hormone therapy, immunotherapy, stem cell transplant, cystoscopy, and breast reconstruction. Most had been involved in a cancer support group (57.9%), and of those, 36.4% were currently attending meetings. Participants had attended a variety of exercise oncology programs, and the majority (78.9%) attended in-person classes prior to COVID-19, ranging from eight weeks to six years. Participation in online exercise oncology programs ranged from several weeks to just over two years, and most were currently attending either ACE or EXCEL online (84.2%).

### 2.4. Procedures

Ethical approval was obtained from the Health Research Ethics Board of Alberta (HREBA.CC-20-0437) on 24 December 2020. A participant advisor was involved throughout the project and consulted about study materials (i.e., demographic questionnaires and interview guides) prior to data collection and about the interpretation of study findings. In line with interpretive description, a theoretical scaffolding document was written at the outset of the study, where the first author identified and wrote about known theoretical, empirical, and practical knowledge to situate the research process and data interpretation [23].

#### 2.4.1. Document Review of Fitness Professional Training Materials

The first author conducted a document review of the online education modules and written resources used in fitness professional training. Following principles for open-ended ethnographic field notes [23], they made notes of their observations pertaining to SS, interpersonal interactions, and supportive behaviours.

#### 2.4.2. Observation of Fitness Professional Training Session

After they complete online modules and readings, fitness professionals attend an online training session on logistics and practical concerns of leading the classes and opportunities for discussion and questions. Sessions are typically four to six hours, but due to the timing of data collection and training session offerings, the first author observed a 3-h modified session. All participants in the training were notified in advance about the observation and that no identifying information would be recorded, and they were invited to contact the first author if they had concerns about being observed so the observation could be rescheduled. No concerns were raised. Ethnographic field notes [25] were collected using a form developed by the research team to prompt the researcher to note information relevant to constructs identified in the theoretical scaffolding and document review related to SS, interpersonal interactions, and barriers to providing or facilitating support. The participating-to-write approach was used, which involves noting initial impressions, significant events, and personal reactions while conducting the observation and participating in the setting [25].

#### 2.4.3. Observations of Online Exercise Classes

Following the training session, instructors in training shadow and moderate classes for 12 weeks with experienced instructors and gradually take on responsibilities for leading the class while receiving feedback from the experienced instructor. If they are deemed competent after 12 weeks, they may instruct their own classes. Observing this shadowing portion of the training was logistically challenging in the online environment, so the next focus of data collection was to observe online exercise oncology classes (*n* = 10). ACE and EXCEL program coordinators assisted with inviting 28 fitness professionals to participate in a class observation and an interview. The first 10 who agreed completed both the observation and interview, while the next five volunteers completed an interview only. A consent form and demographic questionnaire were completed via Qualtrics online survey software. Some observations took place before the fitness professional interviews and some after, depending on scheduling needs. Prior to the observations, all people attending the exercise class received an email similar to the one described in the observations of the training session. Field notes were taken [25], and a form similar to that used in the training observations was used to write observations pertaining to interpersonal interactions, verbal and nonverbal supportive behaviours, and barriers to interacting.

#### 2.4.4. Interviews with Fitness Professionals

Interviews were scheduled with the 15 fitness professionals (*n =* 15). A semi-structured interview guide contained questions about SS training they had received (e.g., did you receive formal training on how to provide SS during exercise classes? Please tell me about what you learned), provision/facilitation of SS (e.g., what activities do you include in your exercise classes that may promote support in the group?), and barriers or challenges they face (e.g., can you tell me about any barriers or challenges you face in trying to facilitate support in the exercise classes?) for both the in person and online exercise oncology contexts, as applicable to the fitness professionals’ experience. The interviews lasted between 29 to 72 min (M = 53 min).

#### 2.4.5. Interviews with Adults Living with and beyond Cancer

Interviews were scheduled with 19 adults living with and beyond cancer (*n =* 19). With the assistance of an ACE program coordinator, an email was sent to participants who had given permission to be contacted for future studies. Volunteers contacted the first author, signed consent forms and completed a demographic questionnaire via Qualtrics, and interviews were scheduled. A semi-structured interview guide inquired about receiving SS (e.g., what kinds of things did people do to support you in the exercise classes?), providing SS (e.g., can you tell me about your experiences of providing SS to others in your exercise classes?), and benefits or barriers to receiving SS (e.g., can you tell me about any barriers or challenges you face in obtaining SS in the exercise classes?) for both in-person and online contexts, as applicable to the participant’s experiences. Interviews lasted 24 to 87 min (M = 55 min).

### 2.5. Data Analysis

Interviews were audio-recorded and transcribed by the first author. Data are identified using codes that specify the data source (training materials [TM], training session observation [TSO], exercise class observation [ECO], fitness professional interview [FPI], participant interview [PI]), exercise class or participant number, and for interviews, the participant’s gender and age. NVivo V.12.0 [26] was used to manage data from all sources. The first author read one transcript, coded text to inductively identify concepts related to the research questions and compared codes and grouped common ideas into subsequent transcripts. Throughout the analysis, constant comparison between data sources, with theoretical scaffolding, and feedback from the study team were sought to challenge and question findings. For example, after the data from each of the fitness professional interviews were analyzed and compared to one another, the codes and themes were then compared to the ones found after analyzing the exercise class observations and the theoretical scaffolding concepts and then shared with the second author for feedback. Inductively derived themes and codes that aligned with concepts in the theoretical scaffolding were named consistent with existing literature. Themes were interpreted in light of the theoretical scaffolding. The first author wrote summaries of the findings from each interview and invited participants to review and provide feedback on the interpretations. A reflexive journal was kept throughout the study about how the researcher affected was affected by the research and to record questions, ideas for themes, and emerging patterns [23].

### 2.6. Quality Criteria

Rigor was addressed using four quality criteria of interpretive description [23], epistemological integrity (i.e., justifiable reasoning from assumptions made to methodological rules applied) was addressed by consulting interpretive description principles when making methodological decisions. Representative credibility (i.e., theoretical claims are coherent within the study sample) was addressed by requesting feedback from participants regarding interpretations of their interviews. Analytic logic (i.e., evidence reported of the researcher’s logic, so interpretations are credible) was attended to by taking notes during data collection, analysis, meetings with the research team, and in a reflexive journal to document discussions and decisions made. Interpretive authority (i.e., striving for trustworthy interpretations by explaining the researchers’ potential bias and experiences) was addressed by writing thoughts, interpretations, and reactions in a reflexivity journal, and the first author discussing and receiving feedback on interpretations from the study team and participant advisor. Variability in participants’ experiences was also shared to ensure multiple experiences were reported.

Because researchers play an active role in constructing knowledge in interpretive description, it is important to describe their positionality. The first author is a white cisgender woman in her mid-20’s who is pursuing graduate education, has been physically active throughout her life, and has watched close friends and family be diagnosed with cancer. The second author is a white cisgender woman in her mid-40’s who has been an academic researcher focusing on social relationship processes in PA contexts for over 15 years and has been physically active throughout her life. The third author is a white cisgender woman in her early 50’s who has been physically active throughout her life and in academia, has been examining exercise behaviour change and the impact on quality of life across chronic disease populations for over 25 years. The fourth author is a white cisgender gay man whose research focuses broadly on equity, diversity, and inclusion in sport and physical activity. He has worked as a fitness instructor for over 20 years and is a former competitive athlete.

## 3. Results

A list of supportive behaviours and social and contextual barriers identified are found in Table 2. These findings were organized into themes (italicized headings with subthemes) that describe the functions those supportive behaviours served and were developed based on results from all sources. A third theme focuses on learning to provide SS in online exercise classes rather than in-person and SS training fitness professionals experienced or would find helpful. Examples from the sources of data are identified in the text using the alphanumeric codes presented in 2.5. Data analysis (e.g., ECO1 refers to exercise class observation number 1).

### 3.1. Creating a Welcoming Environment

#### 3.1.1. Providing a Comfortable Space

Behaviours that created a comfortable space for sharing, seeking social support, and welcoming participants were supportive. Fitness professionals created this space by employing group expectations (e.g., encouraged participants to respect others’ treatment decisions; TM) and connecting with participants (TM; ECO). “I’ll always try to make a connection with all my participants… get to know their cancer diagnosis but outside of that as well. Are they working? Do they have a family?” (FPI7, female, 44 years). Fitness professionals and other exercisers both provided a comfortable space by sharing advice or expressing empathy about cancer and comforting those who were struggling (e.g., instructor checking in with participant who recently lost their spouse before letting others into Zoom room; ECO1). These behaviours align with providing a safe haven from SS theory [8] because they create an understanding, accepting, empathetic, and emotionally safe environment that allows for the expression of negative emotions and vulnerability. While most participants found sharing a common experience with other exercisers was supportive, one participant felt, “There was no connection. There was no common ground other than we have cancer” (PI33, female, 68 years).

Barriers related to participants feeling uncomfortable in the online environment. Fitness professionals were told to expect discomfort with technology (TSO), often offering instruction for navigating and using Zoom, but participants varied in their comfort in the online context. Participants who had connections from in-person classes tended to feel more comfortable interacting online, while many who joined after classes moved online expressed that meeting other exercisers in person may have helped their online interactions feel more natural.

#### 3.1.2. Engaging Participants in Social Interactions

Engaging participants in conversation helped develop relationships and foster cohesion. Engaging with participants and implementing interactive components was emphasized in the fitness professional training. Fitness professionals promoted social time (TSO), facilitated discussions (e.g., by asking questions; ECO), and used language to foster feelings of community (TM), “Good work team!” (ECO12). Fitness professionals and other exercisers becoming actively involved in the conversation (TM and TSO) and going out of their way to involve other participants (e.g., acknowledging things happening on participants’ screens; ECO) was supportive. Verbal interaction is the main form of communication online, so engaging in conversation is critical for initiating relationships. These behaviours were not closely aligned with existing functions in SS theory [8] but may serve a supportive role of engaging and including participants, which may be particularly relevant for initiating new interactions or relationships in contexts where participants may not be close.

Barriers related to not having time or opportunities to engage and challenges to communicating online. Feeling part of the group was also a challenge for new participants, “Prior relationships can dominate the conversations…[They] all have things to talk about, and they’re asking specific things…If it went for half an hour, I’m sure we’d get to that person that’s new, but then it’s time to work out” (FPI2, female, 24 years). Many participants and fitness professionals suggested using breakout rooms, especially at the start of sessions, to build relationships in small groups, reducing pressure on the instructor to lead large group discussions.

#### 3.1.3. Being Open with Others

Helping participants be open and share with the group was supportive. When fitness professionals and other exercisers shared about themselves, it created an environment where being vulnerable was normal. Being open helped the group learn about one another and initiate relationships. For example, some found other exercisers being open about their cancer experiences fostered relationships: “Real life experiences draw us closer, and it is a big support in our own journeys seeing someone else succeed or [say] ‘look my eyebrows are turning white and falling out’” (PI20, female, 60 years). Furthermore, when they remembered things about participants, it showed they were listening and interested (e.g., asking about each other’s dogs; ECO2). These behaviours align with providing a safe haven [8] because they show understanding and reassurance and normalize expressing vulnerability and emotions.

Barriers related to having little opportunity to share and not knowing the others well enough to feel comfortable opening up. Some participants struggled to relate to others because of demographic differences, “You know when you just connect better with similar others? I think because I didn’t see anyone else as a similar other, I had zero support and sometimes felt very uncomfortable” (PI35, female, 24 years). Some participants also had difficulty receiving or providing SS because they did not know one another well, “You have to ask if you need help, and that mostly would come from the instructor. Because you don’t really know the other participants that well” (PI31, female, 59 years). Participants suggested creating a buddy system or having opportunities to interact with a few other exercisers at the time of onboarding. Being open with the group was not emphasized in training, but creating these opportunities and being intentional may be important as participants did not necessarily know each other previously.

#### 3.1.4. Positive and Upbeat

Creating a fun, positive, and upbeat environment was supportive. Fitness professionals and other exercisers who had an energetic or upbeat demeanour (TM and TSO) and laughed or joked throughout the class were supportive (e.g., participant held up ‘out of order’ sign when too tired to keep exercising; ECO6). These behaviours align with nurturing a desire to create or seize opportunities for growth from SS theory [8] by expressing enthusiasm for exercise. Other exercisers created a positive atmosphere by acknowledging their adversity while suggesting positive reframing of their cancer experiences or being role models for living with cancer. These behaviours align with assisting in reframing/redefining adversity as a mechanism for positive change [8] because seeing others reframe their adverse experiences and live well with cancer may help participants reframe their adversity as well.

Barriers included exercisers comparing or critiquing others’ treatment decisions or not being open to exercise corrections or advice from the fitness professional. Role modelling can be a supportive behaviour, but when other people are demonstrating behaviours that the participant may see as unproductive, it can be difficult to be around. It can also be challenging to be a supportive role model when everyone is at different points in their own processes of coping with cancer due to concerns about imposing views that others are not ready for. Overall, participants discussed how the group would try to “find a positive way through things” (PI24, female, 55 years) but felt it was difficult to connect with those who did not share this mindset.

### 3.2. Helping Improve Exercise Ability and Reach Goals

#### 3.2.1. Supporting Mastery

Supporting mastering physical skills and developing mastery (focus on personal improvement, limiting judgements based on comparison with others) was emphasized in training and was frequently experienced as supportive. Fitness professionals provided mastery support by teaching and explaining exercises (TM), wording feedback as what someone can do versus what they should not do (TM), helping goal-set (TM and TSO), ensuring correct execution of exercises (TM and TSO), acknowledging when exercise was done well, and providing feedback or modifications (TM and TSO). “I’ve got arthritic feet…they do the most difficult, the medium, and then the…less weight. So, they’ll make a point of saying ‘okay [name] is this one good for you?” (PI21, female, 57 years). These behaviours align with facilitating preparation for engagement in life opportunities from SS theory [8], because they help develop and recognize PA skills, provide instrumental and informational assistance, and encourage participants to set attainable goals. Fitness professionals and other exercisers suggesting equipment or new types of exercises to try (TSO), celebrating PA goal achievements, and acting as role models and challenging participants to push themselves (TM) was supportive. “If I see somebody doing something in a different way that looks like a slightly harder version of an exercise, then to me, that’s an invitation to challenge myself” (PI25, female, 48 years). These behaviours align with nurturing a desire to create or seize opportunities for growth through expressing enthusiasm and encouraging participants to challenge themselves and facilitating implementation by serving a launching function by celebrating participants’ PA successes to encourage continued engagement [8].

Barriers related to characteristics of the group setting and limitations of interacting online. “Providing feedback… you can’t speak to a specific person. In person, I would be able to go over to someone and discreetly say “hey, why don’t you try this?” or have that conversation without disrupting everyone else” (FPI14, male, 27 years). Comparing to other exercisers was a barrier experienced by some, but most participants did not compare or feel pressured to exercise at a certain level. “Because [online] I’m only focused on the instructor… I don’t have to worry about who’s standing next to me” (PI23, male, 66 years). The fitness professional training also emphasized avoiding comparing participants to one another (TM).

#### 3.2.2. Encouraging

Encouraged PA during and beyond the class was supportive. The fitness professional training discussed encouragement, but these findings provide examples of how and what can be encouraged. Fitness professionals drew positive attention to participants, which facilitates preparation for engagement in life opportunities in SS theory [8]. Fitness professionals also motivated participants to keep exercising throughout the class with high fives to the screen (TSO) and encouraging comments, “Hang in there with those monster walks, feel that burn!” (ECO 3). Fitness professionals and other exercisers encouraged engaging in PA outside of the class (TSO), coming back each week (TSO), and provided individualized and general encouragement (TM and TSO). These behaviours serve a launching function [8] by building confidence and creating a safe and secure base to start exploring other exercise opportunities. While some participants voiced their preference for individualized encouragement, no barriers to receiving or providing encouragement were identified. This may reflect the high quality of the training, as most participants felt receiving encouragement was supportive, and all fitness professionals recognized the importance of providing encouragement.

#### 3.2.3. Supporting Autonomy

Giving freedom to program participants to control and choose their PA behaviour was experienced as supportive. Providing autonomy support was emphasized in the fitness professional training through presenting choices such as exercise and intensity (TM) and encouraging participants to listen to their bodies and modify as needed (TM and TSO). “He says, ‘Okay we’re going to work on the arms’ and you’ll find three, four, five, six other people doing something different than what he’s suggesting, but all the right thing for the same muscles” (PI34, male, 68 years). Fitness professionals also encouraged participants to think about why they came and what they would like to get out of class (TM and TSO), asked for their feedback, and encouraged them to interrupt and ask questions, “Feel free to come off mute and interrupt me if anything changes” (ECO11). These behaviours align with autonomy support from self-determination theory, referring to being the source of one’s own behaviour, which can be influenced or requested by others (in this case, by fitness professionals) [27]. It may also serve as a launching function [8] because participants are free to explore and choose exercises consistent with their preferences and ideal self. As a result of being online, participants felt more comfortable exercising on their own and more responsible for their exercise quality, “They cannot see subtle aspects of your exercise, so I had to uptake the responsibility for doing the exercise mechanically as correct as possible” (PI19, male, 70 years).

Barriers related to fitness professionals not having as many opportunities to allow participants to control their behaviour in the online environment. Fitness professionals felt participants were able to approach them more easily and often in person. While most fitness professionals were observed providing modifications and variations for each exercise in the online environment, they still felt participants had more opportunities to discuss and try different exercises in person. Furthermore, fitness professionals were unable to provide opportunities for participants to choose who they wanted to talk to in the online environment, limiting autonomy.

#### 3.2.4. Participating Together

Other exercisers enabling their participation in exercise was supportive. The fitness professional training did not focus on fostering these behaviours among participants explicitly. Many participants did not have strong connections with other exercisers in the class but found it supportive when they exercised with participants consistently (inside or outside class) or were committed. “Just the fact they showed up, because I can see them… And so, the sense of participation is something that makes me feel supported” (PI17, female, 68 years). When participants had connections to instructors or other exercisers, they wanted to come to class and see the group. These behaviours did not align with the functions of SS theory [8] but identified a new SS function more pertinent to new or less intimate relationships.

Barriers pertained to having no social connections with others in the class and others not being committed themselves, making it easier to lose motivation and skip classes or feel less committed. “If they weren’t there and they didn’t make the effort to turn up, I wouldn’t be there” (PI28, female, 74 years). Some participants felt the minimal social connections they had with others were not enough of a motivator to come to class. Having social connections with others and seeing they are committed may be critical for participants wanting to attend the classes, and having more substantive conversations may help develop stronger connections.

#### 3.2.5. Informational Support

Behaviours that provided information and resources about PA to participants were supportive. Fitness professionals were trained to share information about the benefits of exercise (TM and TSO), which aligns with nurturing a desire to create or seize opportunities for growth from SS theory [8] because they are communicating the benefits of pursuing exercise. Fitness professionals sent information about classes weekly (TSO), and both fitness professionals and other exercisers were supportive when they suggested activities or resources (e.g., YouTube videos with workouts (ECO10) to use to continue exercising between classes. These behaviours align with facilitating preparation for engagement in life opportunities [8]. The exercise program also provided informational webinars and movement challenges to help participants learn more about exercise, and fitness professionals were encouraged to bring up these topics during class to facilitate discussion between participants (TM and TSO). “Information topics…at the beginning of the week, we pick a couple minutes to chat about them…go in depth and ask about their experience and how they can put that into their lives” (FPI8, female, 36 years). There were no barriers to receiving or providing informational support observed or discussed in interviews. Most participants praised the program and instructors for the abundant information provided. Furthermore, all fitness professionals recognized the importance of disseminating helpful resources and their own knowledge.

### 3.3. Learning to Provide and Facilitate Support Online

#### 3.3.1. Delivering Social Support in the Online Group Exercise Class

Fitness professionals felt they had a larger role online because they now did the entire workout with participants, led discussions, were the main person talking throughout class and had to intentionally and consciously provide and facilitate SS. “[In person] that social support piece is already there, you’re already all in the same room together, it’s more organic conversations before and after class…Online you have to implement strategies to have social support” (FPI6, female, 23 years). Some fitness professionals struggled to provide SS while running the class and not being physically present with participants. They also felt communicating or delegating to the moderator in the online environment was difficult (TSO). “[In person] you can ask them ‘Oh just go over and help that person, give them a little bit more feedback’. But now you can’t really communicate with your moderator unless your typing…[But] you want to be watching what’s going on” (FPI5, female, 24 years).

To help address these challenges, the program made sure two fitness professionals (i.e., instructor and moderator) ran the classes, which helped share responsibilities and made participants feel supported. “There’s two of them, so one that keeps track and watches to see if anybody needs something, and the other one does the exercises. [Or] sometimes one does the floor exercises, and the other does the more difficult ones” (PI27, female, 68 years). There was a discussion in the training session about the importance of fitness professionals building rapport with one another to be able to work together seamlessly (TSO). Some behaviours discussed in the “engaging participants in social interactions” subtheme were suggested to fitness professionals to help them deliver SS online. For example, fitness professionals explained that asking a question of the day to the group provided more opportunities for participants to learn about one another, which helped to form connections and facilitate communication among participants, reducing the reliance on the fitness professional to maintain the conversation.

#### 3.3.2. Social Support Training Needs

Most fitness professionals identified that the training talked about different types of SS and the importance of SS for exercise adherence but felt there could be more emphasis on how to provide these forms of SS. Many fitness professionals did not initially receive training on how to provide SS in the online environment because the transition to online happened quickly when the pandemic began. However, they had in-services to discuss challenges, which often included topics related to SS. Many fitness professionals found shadowing or moderating exercise classes of experienced instructors was an effective way to learn how to provide and facilitate SS. They also discussed the intentionality that is needed for providing SS online, which they sometimes thought felt forced compared to the in-person environment. They acknowledged the importance of adapting or tailoring different SS techniques given the unique needs of different classes and individuals, with one moderator wishing they received additional training about adapting exercise to participants’ specific drug and treatment side effects. They felt it was particularly challenging to support someone through difficult life circumstances such as grief or health concerns because privacy is difficult to maintain in the online group setting, and not being physically in the same place can affect feelings of connection and embodied presence with the other person. All fitness professionals believed that more formal training would be beneficial to provide a framework for future instructors experiencing online instruction for the first time.

Fitness professionals want to be provided with specific examples or strategies in training for how to provide and facilitate SS online. “Knowing how to implement it effectively, but also having a variety of different ways to implement it so that other people will maybe feel inclined to connect socially” (FPI11, female, 25 years). Some fitness professionals wished they had a “Toolkit” (FPI15, male, 24 years) of questions or topics to help in situations where they are struggling to start a conversation. “When you’re in the moment and you’re put on the spot, ‘What do I say?’ ‘What do I talk about?’ So, I think if there were some ideas of what you could get them talking about” (FPI4, female, 23 years). Fitness professionals want information about SS strategies to help them connect with their participants and facilitate connection among participants online.

## 4. Discussion

The purpose of this study was to examine the current practices for training fitness professionals to provide and facilitate SS, experiences adults living with and beyond cancer have with SS, and behaviours and barriers for providing and obtaining SS in online exercise oncology classes. This study identified supportive behaviours that address creating a welcoming environment and helping improve exercise ability and reach goals, some of which align with prior theory and some which may be particularly important in situations where participants do not already have a close bond. Barriers related to the context, ability to interact, and provision of exercise instruction that prevented or hindered SS online. Additionally, fitness professionals value SS and want more training for how they can provide and facilitate SS.

Behaviours that served the function of providing a comfortable space, being open with others, being positive and upbeat, supporting mastery, encouraging, supporting autonomy, and providing informational support aligned with functions from SS theory [8]. Some supportive behaviours identified that align with functions in SS theory have also been found in prior literature [1], however, functions that our findings aligned with may be particularly relevant to newer relationships in the online context. Many of these behaviours can be provided by other exercisers or fitness professionals without a strong relational foundation being in place but may be critical for creating a comfortable and encouraging online environment. While some participants may have pre-existing close relationships, or develop them over time, many of the relationships in these contexts are not close and long-established. Therefore, our findings had fewer behaviours that may be more inceptive of well-established, close relationships. The current study did not identify supportive behaviours that aligned with providing fortification, assisting in the reconstruction process, and providing perceptual assistance in the viewing of life opportunities [8]. These functions help to develop or recognize talents/abilities and problem-solve to cope with adversity, as well as to focus on positive aspects of opportunities instead of the potential for failure. Therefore, they may be more pertinent for closer friendships or relationships outside of the exercise class (e.g., family). Future research should examine closer relationships in this context to see if supportive behaviours pertinent to these three functions from SS theory [8] are evident.

The SS functions engaging participants in social interactions, participating together, and some SS behaviours in being open with others did not align with SS theory [8]. This may be because SS theory was developed with well-established relationships, and the SS functions we identified may be critical for fostering newer relationships. The relationships in the group exercise oncology context are often loose social ties that provide camaraderie to participants [1]. Meeting other exercisers and instructors and being around them repeatedly over time can be supportive [1] and they were found to be supportive in the function of engaging participants in social interactions. In the general population and with adults living with and beyond cancer, building trust, sharing with other exercisers and instructors, and sharing PA experiences with others can provide accountability and motivation [1,28]. These supportive functions and behaviours are not new to the literature, but considering their role in online exercise is a novel way to look at them, as these supportive functions may be geared toward helping establish relationships or obtain support from those with weaker social ties. The opportunities to connect in the online exercise context are often limited and feel forced [28]. Therefore, these SS functions may be critical to facilitate and implement in this context.

There are barriers to providing, facilitating, and receiving SS in the online group exercise context. Contextual barriers related to the group or online context and participants could be intimidated when joining a group that had established relationships prior [1]. In the online context, comparing to others and being self-conscious, for example, because of age differences [28] and being unfamiliar with the Zoom platform were barriers [17]. There were barriers impacting interaction and socialization in the online environment and recent studies with various populations have also found the social aspects of these classes were impacted due to participants not being physically together and having limited opportunities to connect [17,28,29]. Not having these social connections with the group impacts participants’ motivation. There were also barriers to receiving and providing exercise instruction in the online environment, including difficulties receiving personalized feedback from instructors [17], and challenges for fitness professionals to see participants form and provide public feedback [28].

Fitness professionals valued SS in their exercise classes and welcomed training or evidence-based practices. They wanted a framework with applied examples or behaviours for providing and facilitating SS. This is a practical issue in the SS literature and one of the main reasons why Feeney and Collins developed their SS theory [8] to focus on supportive behaviours. There is a need for fitness professionals to learn how to facilitate interaction in their exercise classes, especially in the online environment where it is often the fitness professional leading discussions and talking to individuals rather than participant-to-participant interactions [28]. Many participants in the current study felt they had a strong relationship with the instructor but not the other exercisers. Participants also have different SS needs and preferences, so communicating with them to learn and understand their needs is critical to providing high-quality support.

Limitations include that although this study included an observation of a staff training session, the first author was unable to attend a full (4–6 h) day session and observe fitness professionals being trained to instruct within the exercise classes. Both samples (adults living with and beyond cancer and fitness professionals) were primarily female-identified, white, and tended to have moderate to high incomes, so they are not representative of the population. Additionally, the adults living with and beyond cancer and fitness professionals recruited may have had overly good experiences with the exercise program and value SS, and therefore were more likely to volunteer for this study. Future studies should use recruitment strategies that may reach a more diverse sample of both populations. A strength of the current study was the involvement of a participant advisor. Their feedback provided confirmation that the final report captured the experiences of participants in the online exercise oncology classes, has the potential to be beneficial for improving SS in the classes, and is understandable to the reader.

Practical implications include fitness professionals being trained to use behaviours that foster feeling comfortable being open and vulnerable, sharing about their lives, and engaging with one another. Training should help instructors facilitate SS by providing quality exercise instruction and developing relationships with participants through social interactions. Training should also encourage the facilitation of interactions between participants by finding commonalities and facilitating communication. Facilitated discussions can feel forced but may be important at the start of the exercise program to help initiate connections. It is also important that fitness professionals do not feel overwhelmed with the added responsibility of providing and facilitating SS throughout the online class. To help with this, participants can be encouraged to provide SS to one another, and the program can provide resources, learning topics, and/or webinars regarding behaviours that can be used to provide SS. Theoretical implications include the findings identifying supportive functions and behaviours that are relevant in the online group exercise oncology context where relationships may be newer or less intimate than the established relationships that were the focus of Feeney and Collins’ work developing their SS theory [8]. It may be important for future research to examine and add supportive functions that consider these less intimate relationships.

## 5. Conclusions

This study adds to the literature by identifying socially supportive functions and behaviours within the online group exercise oncology context. Supportive functions, which may be particularly important in newer relationships, were identified that align with SS functions from Feeney and Collins’ theory [8]. Additionally, some of our findings were not aligned with SS theory [8], which may be important for developing connections initially or fostering newer relationships. These findings identify supportive behaviours and barriers to providing and obtaining support that can aid exercise program developers in structuring their online classes. Furthermore, these findings can be used to inform fitness professionals’ training and practice by intentionally fostering support by considering participants’ wants and needs, acknowledging the value of shared and common experiences among participants, and integrating support practices during exercise instruction in the context of a welcoming and caring environment. SS may enhance participant experiences by positively impacting exercise adherence and the many potential beneficial outcomes of online group exercise oncology classes. Future research should focus on the development of strategies and training for fitness professionals to foster SS. Further research should also examine the effects on participant well-being and exercise adherence after implementing supportive behaviours and strategies into fitness professionals’ training.

## Figures and Tables

**Table 1:** Participant Characteristics.

	Fitness Professionals		Adults Living with and beyond Cancer	
	*n*	*M* (*SD*)	*n*	*M* (*SD*)
Age	15	27.6 (7.5)	19	59.3 (11.0)
	*n*	%	*n*	%
Gender Identity *				
Female	12	80.00	14	73.68
Male	3	20.00	5	26.32
Sexual Orientation				
Heterosexual/straight	13	86.67	14	73.68
Gay/lesbian	1	6.67	1	5.26
Did not respond	1	6.67	4	21.05
Race/Ethnicity				
White	11	73.33	16	84.21
South Asian	1	6.67	0	0.00
South Asian and White	1	6.67	0	0.00
Metis	1	6.67	0	0.00
Chinese	0	0.00	1	5.26
Pakistani	0	0.00	1	5.26
Did not respond	1	6.67	1	5.26
Working Status				
Retired	0	0.00	31	43.06
Disability or sick leave	0	0.00	14	19.44
Working/studying full-time	13	86.67	9	12.50
Homemaker or stay-at-home parent	0	0.00	8	11.59
Working/studying part-time	2	13.33	7	10.14
Education				
High school diploma	2	13.33	1	5.26
Some post-secondary	1	6.67	1	5.26
College or technical degree/diploma	0	0.00	6	31.58
University undergraduate degree	6	40.00	7	36.84
Post-graduate degree	6	40.00	4	21.05
Household annual income				
Less than $5000	1	6.67	0	0.00
$15,000 to $19,999	1	6.67	0	0.00
$25,000 to $29,999	0	0.00	1	5.26
$30,000 to $39,999	3	20.00	0	0.00
$40,000 to $59,999	1	6.67	0	0.00
$60,000 to $79,999	2	13.33	1	5.26
$80,000 or over	6	40.00	11	57.89
Prefer not to answer	1	6.67	6	31.58

* Gender identity was self-identified as “female” and “male” by participants.

**Table 2:** Supportive behaviours and social and contextual barriers.

Supportive Functions	Supportive Behaviours	Source of Supportive Behaviours	Barriers to Supportive Functions
Creating a welcoming environment			
Providing a comfortable space	Encourage exercisers to respect each other’s treatment decisions	FP	Cancer commonality is not always enough to form relationships Not knowing other participants in person Familiarity or comfortability with technology
	Make themselves available as a support person	FP	
	Ask participants what SS they want	FP	
	Ask participants what support they have at home while exercising	FP	
	Get to know the group	FP	
	Relate to participants about being sore and tired during exercise	FP	
	Help participants learn technology	FP	
	Listen or comfort if needed	FP and OE	
	Share resources, advice about cancer	FP and OE	
	Discuss and empathize with cancer experience	FP and OE	
Engaging participants in social interactions	Encourage participants to stay unmuted	FP	Unable to meet in person outside of class due to COVID Fitness professional and/or participants do not come to class early or stay late Little opportunity to socialize or provide support because of online format Cannot use chat function while exercising Interaction can be disruptive FP or OE do not interact with the group Bring up divisive topics of conversation, might not be inclusive Publicly asking participants to speak might make them uncomfortable Difficult to make connections between new and experienced participants Hard for new participants to join in conversation Difficult to feel like a group or team Embodied social interaction is different (e.g., difficult to read nonverbal cues, cannot feel the presence of others) Can only have group conversation, less opportunity for one-on-one interactions Need to take turns talking Not enough time for all to share Difficult to build rapport, start and moderate conversations, facilitate cross-talk Must lead the discussion and be the main person talking, do not always feel prepared to do so
	Encourage and give time before and after class to socialize and ask questions	FP	
	Use language to foster group or community	FP	
	Encourage seeking SS	FP	
	Interactive warm-up, exercises, activities	FP	
	Ask a question of the day	FP	
	Call on participants to speak	FP	
	Acknowledge things happening on screen	FP and OE	
	Ask questions and engage in group conversation	FP and OE	
	Facilitate or encourage connection outside of class	FP and OE	
	Introduce new participants and include them in conversation and activities	FP and OE	
	Interact during the class	FP and OE	
	Greet, check-in, use names	FP and OE	
Being open with others	Remember things about each other, follow-up	FP and OE	Not comfortable opening up because it is difficult getting to know personalities, interests, and personal lives Not comfortable opening up because one does not think group wants to hear about one’s problems Difficult to recognize if others are struggling and express support Little opportunity to talk about cancer Difficult to connect to others if one does not see one’s own culture or representation in the group
	Share stories, personal life, interests	FP and OE	
	Share about cancer, treatments	OE	
Positive and upbeat	Plays energetic, upbeat music	FP	OE comparing or critiquing others’ treatment decisions OE not being open to FP exercise corrections or advice
	Upbeat, energetic, personable	FP and OE	
	Celebrate or compliment each other	FP and OE	
	Have fun, laugh, and joke	FP and OE	
	Acknowledge their adversity while suggesting positive reframing of cancer experience	OE	
	Role model living a happy, positive life despite cancer	OE	
	Encourage, share kind words with those going through treatment	OE	
Helping improve exercise ability and reach goals			
Supporting mastery	Explain exercises in multiple ways to aid understanding	FP	Difficult for FP to see participants FP cannot physically correct form Cannot provide feedback privately Difficult to individualize for each person Feel judged or compared to others Those with higher skill level or physical ability feel they are showing off if they push themselves
	Word feedback as what they can do versus what they should not do	FP	
	Check-in during exercise, re-demonstrate if needed	FP	
	Ask if anyone has questions	FP	
	Provide high-quality workouts	FP	
	Provide individualized feedback	FP	
	Provide modifications	FP	
	Ask about injuries and side effects of each participant so they can be accommodated	FP	
	Reassure that nobody is watching participants exercise, nobody is there to judge	FP	
	Express pride in participants who came to class	FP	
	Acknowledge when exercise is done well	FP	
	Help to goal-set	FP	
	Celebrate when PA goals are achieved	FP and OE	
	Suggest equipment for participants to use or try	FP and OE	
	Role model, challenge, or push each other to exercise	FP and OE	
Encouraging	Draw positive attention to participants	FP	
	Provide motivational talk	FP	
	High five the screen	FP	
	Remind to not be discouraged when physical ability is not the same as before treatment	FP	
	Encourage or help engage in other PA	FP and OE	
	Encourage to come back each week	FP and OE	
	Provide individualized and general encouragement	FP and OE	
Supporting autonomy	Encourage to listen to body, modify or adjust to accommodate needs	FP	Cannot choose who to talk to during exercise Participants are not always aware they can choose whatever exercises they want
	Do not push when participants share they are not feeling well	FP	
	Encourage to progress at own pace	FP	
	Ask what they want to get out of class	FP	
	Encourage to interrupt if needed	FP	
	Give options to choose exercises	FP	
	Invite suggestions, feedback	FP	
	Incorporates participant music suggestions	FP	
Participating together	Commit, show-up to class	OE	Not having connections with others makes it easier to skip class Social connections do not motivate participants to attend When others do not commit, participants do not want to commit
	Exercise together outside of class	OE	
	Join the same class to stay together	OE	
Informational support	Communicate the benefits of exercise	FP	
	Email exercises information about class	FP	
	Share exercise circuit instructions in the chat	FP	
	Suggest resources or activities to do or use between classes	FP and OE	

Note: FP = Fitness professionals; OE = Other exercisers.

## Data Availability

The participants of this study did not give written consent for their data to be shared publicly, so due to the sensitive nature of the research supporting data is not available.
